# Optical probe for surface and subsurface defects induced by ion bombardment

**DOI:** 10.1002/pssr.201307088

**Published:** 2013-03-05

**Authors:** L D Sun, M Hohage, P Zeppenfeld

**Affiliations:** Institute of Experimental Physics, Johannes Kepler University LinzAltenbergerstr. 69, 4040 Linz, Austria

**Keywords:** metal surfaces, ion bombardment, reflectance difference spectroscopy, optical anisotropy, surface defects, bulk defects

## Abstract

We demonstrate that reflectance difference spectroscopy (RDS) is sensitive to defects induced by ion bombardment, located either in the topmost layer or in the subsurface region. Most importantly, these two kinds of defects can be spectrally discriminated, since the corresponding signatures in the RD spectrum arise from perturbations of different types of electronic states: The defects in the topmost surface layer mainly lead to a quenching of the optical anisotropy related to surface states, whereas the subsurface defects strongly affect the optical anisotropy originating from transitions between surface-modified bulk electronic states. Consequently, RDS can be used to simultaneously monitor the defects in the topmost surface layer and in the subsurface region *in-situ* during ion bombardment and thermal annealing.

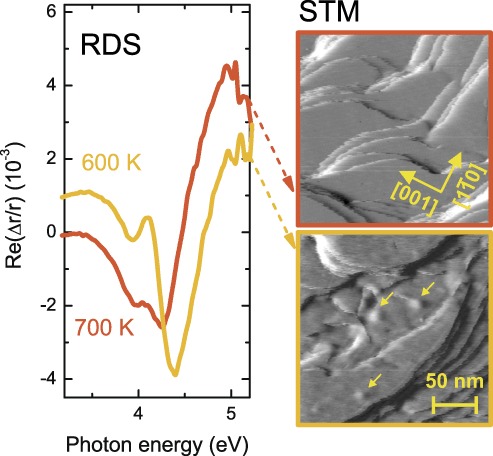

Characteristic RD spectra and the corresponding STM images for a Cu(110) substrate before and after healing of the subsurface defects.

## 1 Introduction

Rare gas ion bombardment and subsequent thermal annealing is routinely used to prepare clean and smooth surfaces for surface studies and subsequent thin film growth. Ion bombardment of a single-crystal metal substrate not only induces defects in the topmost surface layers and changes the surface morphology 1–3, it also creates bulk defects in the subsurface region which modify the strain field in the near-surface region 4–8. Both kinds of defects have a strong impact on the surface properties. It is thus desirable to monitor the morphology of the topmost surface and the concentration of defects in the near-surface region simultaneously and *in-situ* during ion bombardment and subsequent thermal annealing.

Reflectance difference spectroscopy (RDS) measures the difference of the normal-incidence reflectance for two perpendicular orientations of the polarization vector as a function of photon energy 9. In this study, we apply RDS as a sensitive probe to defects formed in the topmost surface layer as well as in the subsurface region during ion bombardment. The optical discrimination is based on the fact that the defect-induced perturbation of the optical anisotropy is different for the defects in the topmost layer (surface defects) and in the subsurface region (bulk defects). Surface defects mainly perturb the surface states of metal substrates which leads to strong quenching of the related RDS signal. On the other hand, subsurface defects influence the bulk electronic structure in the near-surface region, with a pronounced effect on the corresponding optical anisotropy. In fact, the sensitivity of RDS to thermally and ion-induced defects on noble metal surfaces has been recognized by several authors in the past 10–15. In particular, a sensitive dependence of the surface-states-related RD signal on the surface defect concentration and distribution has been elucidated 15–18. Here, we will demonstrate the complementary correlation between the optical signature originating from surface-modified bulk electronic states and the presence of bulk defects in the near-surface region. Based on a thorough understanding of the origin of the RD spectrum, one can use the spectral signatures arising from optical transitions between surface states and surface-modified bulk states as fingerprints of surface and bulk defects, respectively, in order to simultaneously monitor the formation and healing of both types of defects during ion bombardment and subsequent annealing.

## 2 Experiment

The experiments have been carried out in a UHV chamber with a base pressure of 1 × 10^–10^ mbar. The substrate can be heated by electron beam bombardment from the backside and cooled down to 20 K with a continuous flow liquid He cryostat. The Cu(110) surface was cleaned by repeated cycles of 900 eV Ar^+^ ion sputtering and subsequent annealing to 800 K. The ion bombardment and subsequent annealing is monitored *in* -*situ* using a photoelastic modulator (PEM)-based RD spectrometer 19, 20. The normalized difference in reflectivity 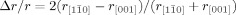 is recorded either in spectroscopic mode over a photon energy range between 1.5 and 5.5 eV or by recording transients where the 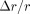 signal at a selected photon energy is measured as a function of time.

## 3 Results and discussion

**Figure 1:**
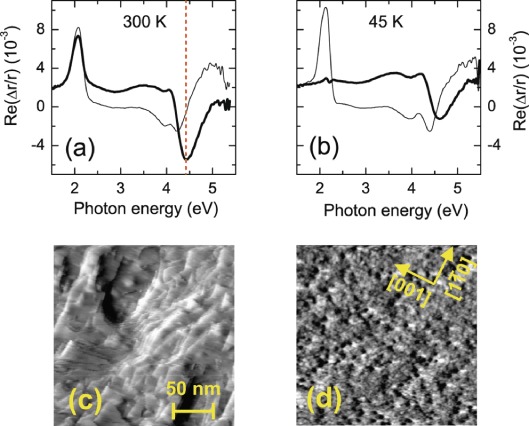
(a) and (b) Real part of the RD spectra recorded before (thin lines) and after bombardment (thick lines) with 1000 eV Ar^+^ at room temperature (one hour) and at 45 K (30 min), respectively. (c) and (d) STM images of the ion bombarded surfaces in (a) and (b), respectively.

Figure 1 illustrates the different effect of ion bombardment of the Cu(110) substrate at room temperature and low temperature (45 K). As reported previously, the optical anisotropy of Cu(110) shows two main features, namely, a positive peak at 2.1 eV and a negative signature around 4.3 eV 9. The response of these two features to the ion bombardment is obviously quite different. Similar to what has been reported earlier 12, bombarding the Cu(110) substrate at room temperature strongly affects the 4.3 eV feature, whereas the 2.1 eV signal decreases only slightly. At 45 K, however, the 2.1 eV peak is completely quenched, as also reported recently by Isted et al. 15.

The completely different behavior of the RDS signal at 2.1 eV can be related to the surface morphology induced by ion bombardment at the corresponding temperatures. As can be seen in Fig. 1(c) and (d), respectively, Ar^+^ ion bombardment creates surface vacancies and adatoms, which are practically immobile at 45 K (d) but can arrange into larger, compact structures at room temperature (c). In spite of the high step density, the surface in Fig. 1(c) exhibits sizable terraces with straight step edges oriented along the main crystallographic directions. In contrast, after bombardment at 45 K, the surface is totally rough at the nanometer scale and appears structurally isotropic 15. The different surface morphology is responsible for the different behaviour of the RD signal at 2.1 eV. In fact, the main contribution to the optical anisotropy at 2.1 eV stems from optical transitions between an occupied and an unoccupied surface state located at the 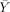 symmetry point of the surface Brillouin zone 13, 21. The surface defects influence the surface state transition in two different ways: (i) the suppression or depopulation of the surface state in the vicinity of defects and (ii) the loss of anisotropy (depolarization) of the surface states over large areas due to scattering of the surface state electrons by surface defects 16, 17. The first effect is spatially rather localized, whereas the second one is quite extended and leads to a very efficient quenching of the optical anisotropy at 2.1 eV 16, 17. According to Fig. 1(c), the surface defects after Ar^+^ ion bombardment on Cu(110) at room temperature, i.e., step edges and dislocation lines, are mostly orientated along the high-symmetry directions ([001] and 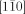) of the surface. The depolarization effect of such kind of defects is negligible 17. The decrease of optical anisotropy in this case is dominated by the local modification of the surface electronic states in the vicinity of the steps and is thus rather limited. In contrast, after ion bombardment at 45 K, the surface is full of point defects, and terraces can no longer be discerned. On this kind of surface, the surface state cannot survive, not to mention its anisotropy. Therefore, the optical anisotropy at 2.1 eV is a sensitive probe of the density and topology of surface defects on the Cu(110) surface.

In contrast to the optical anisotropy at 2.1 eV, the RD signal around 4.3 V is modified in a very similar way upon ion bombardment at different temperatures (see Fig. 1(a) and (b)). In this energy range, the RD spectra after ion bombardment have the same line shape, while the amplitude depends on temperature and Ar^+^ ion fluence. The optical anisotropy around 4.3 eV is attributed to surface modified interband transitions 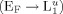 in the vicinity of the high symmetry point L of bulk copper 22. In particular, 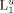 is an s-like electron band and it is known to be very sensitive to strain 23. As a result, the RDS signal at 4.3 eV is extremely sensitive to the strain field in the near-surface region 24, 25. The change of the RD signal at 4.3 eV thus indicates that the ion bombardment induces a measurable strain field at or below the surface. In fact, after room temperature ion bombardment, some broad, shallow protrusions can be discerned (see Fig. 1(c)) indicating the presence of Ar gas bubbles in the subsurface region 4, 8. It has been demonstrated that the surface layers around these gas bubbles are considerably and inhomogeneously stressed 7. Besides, other bulk defects like vacancy clusters and interstitial clusters can be created by ion bombardment and are stable in the Cu bulk even above room temperature 6, 26. These bulk defects introduce inhomogeneous strain fields that extend up to the anisotropic surface, thus affecting the RD signal at 4.3 eV 24.

**Figure 2:**
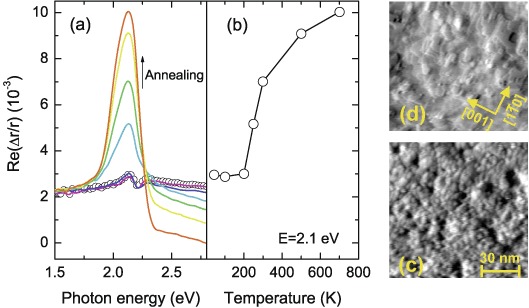
(a) Real part of the RD spectrum after 30 min bombardment at 45 K (open circles) and annealing to 100 K, 200 K, 250 K, 300 K, 500 K, and 700 K. (b) Change of the RD intensity at 2.13 eV upon stepwise annealing. (c) and (d) STM images recorded after annealing at 200 K and 250 K, respectively.

The creation or annealing of surface and subsurface defects can now be investigated independently by monitoring the RD signals at 2.1 eV and 4.3 eV, respectively. As an example, Fig. 2(a) shows the RDS spectra around 2.1 eV recorded at 12 K after successive annealing steps of the Cu(110) surface after the ion bombardment at 45 K in Fig. 1(b) and (d). The 2.1 eV peak starts to recover after heating to 250 K, indicating the onset of the annealing of surface defects. Indeed, the STM images recorded after annealing to 200 K and 250 K in Fig. 2(c) and (d), respectively, confirm that terraces with an appreciable size and straight step edges are only formed after annealing to 250 K. This onset temperature for the annealing of the small structures fits nicely with the kinetic barriers calculated for Cu(110) 27.

**Figure 3:**
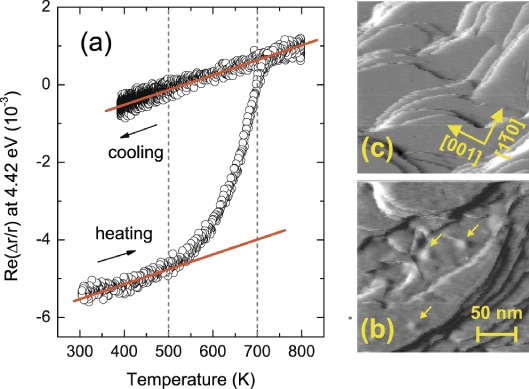
(a) Real part of the RD signal at 4.42 eV (the energy position marked by dashed line in Fig. 1a) recorded during annealing of a Cu(110) substrate which has been bombarded by 1000 eV Ar^+^ for one hour at room temperature. (b) and (c) STM images recorded at room temperature after the substrate has been annealed at 600 K and 700 K, respectively.

In another experiment, the Cu(110) surface was bombarded with 1000 eV Ar^+^ ions for one hour at room temperature and then heated up to 800 K. In this case, the RD signal at 4.42 eV was monitored as a function of temperature and is plotted in Fig. 3(a). To remove the temperature effect (i.e., variation of the RD signal due to the change of temperature) 22, the RD signal was recorded both during heating and cooling the Cu(110) sample. The linear variation of the RD signal upon cooling is a temperature effect due to the shift of the 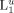 band as a function of substrate temperature 22. Consequently, the identical slope of the RD signal during the initial stage of heating can be assigned to the same temperature effect. The real recovery of the RD signal that is related to the annealing of the subsurface defects only sets in at around 500 K. Above 700 K, the RD transient at 4.42 eV recorded during heating perfectly overlaps with the cooling curve, indicating the complete annealing of bulk defects in the relevant near-surface region. This conclusion is confirmed by STM experiments depicted in Fig. 3(b) and (c): the surface protrusions induced by the Ar gas bubbles can still be discerned in the STM image recorded after annealing to 600 K (marked by arrows in Fig. 3(b)) but have completely disappeared after annealing to 700 K (see Fig. 3(c)). Again, the temperature range in which the RD signal is recovered nicely fits with the known annealing process of bulk defects in copper 6, 26.

## 4 Conclusion

In conclusion, we have shown that reflectance difference spectroscopy (RDS) allows to spectrally discriminate between different types of defects induced by Ar^+^ bombardment of a Cu(110) surface. By measuring the optical anisotropy originating from transitions between surface states and those arising from surface-modified bulk electronic states, we have monitored *in* -*situ* the formation and healing of defects at the surface and in the subsurface region, respectively.
